# Supplementary material for: Longitudinal changes in [18F]FDG PET brain metabolism as a prognostic marker in autoimmune encephalitis
Source: Eur J Nucl Med Mol Imaging. 2025 Aug 25;53(2):1183–97. doi: 10.1007/s00259-025-07526-2 (PMC12830435; doi:10.1007/s00259-025-07526-2)
Supplement: Supplementary file 1 — (DOCX 6.41 MB) [file 259_2025_7526_MOESM1_ESM.docx]

**Supplementary Table 1s.**

**CASE and mRS scores at baseline (BS) and follow-up (FU) for each AE type**

| Auto-antibodies | CASE  (mean±SD) | | mRS  (mean±SD) | |
| --- | --- | --- | --- | --- |
|  | BS | FU | BS | FU |
| Anti-NMDAR (n=4) | 8.0±3.2 | 2.3±1.7 | 3.8±1.3 | 1.5±1.3 |
| Anti-LGI1 (n=6) | 3.9±2.1 | 2.0±1.5 | 3.1±1.0 | 1.5±1.2 |
| Anti-CASPR2 (n=1) | 1.0 | 2.0 | 2.0 | 3.0 |
| Anti-LGI1 and CASPR2 (n=1) | 3.0 | 3.0 | 3.0 | 2.0 |
| Onconeural (n=3) | 3.0±1.0 | 3.3±1.5 | 2.3±1.5 | 3.3±2.1 |
| Seronegative (n=7) | 3.9±2.3 | 2.9±1.7 | 3.3±1.4 | 2.0±1.1 |

**Supplementary Table 2s. Regions with significant metabolic differences between BS and CTR**

| **Hypermetabolic areas at BS relative to CTR** | | | | | | | |
| --- | --- | --- | --- | --- | --- | --- | --- |
| **Cluster p (FEW-corr)** | **Cluster extension (number of voxel)** | **Cluster p (unc)** | **Cluster peaks coordinates**  **x y z** | | | **Cortical region** | **BA** |
| 0 | 3338 | 0 | -4.44 | -61.79 | -35.88 | L cerebellum | **-** |
|  |  |  | -0.82 | -56.91 | -28.15 | R cerebellum | **-** |
|  |  |  | 6.6 | -60.5 | -30.17 | R cerebellum | **-** |
|  |  |  | -13.61 | -57.49 | -41.04 | L cerebellum | - |
|  |  |  | -15.41 | -47.99 | -41.97 | L cerebellum | - |
|  |  |  | -13.57 | -51.73 | -42-29 | L cerebellum | - |
|  |  |  | -2.77 | -52.18 | -18.73 | L cerebellum | - |
|  |  |  | 17.98 | -43.91 | -46.42 | R cerebellum | **-** |
|  |  |  | 15.87 | -54.96 | -29.49 | R cerebellum | **-** |
|  |  |  | 14.22 | -47.97 | -43.27 | R cerebellum | - |
|  |  |  | 23.43 | -37.36 | -36.7 | R cerebellum | - |
|  |  |  | -15.62 | -47.69 | -25.73 | L cerebellum | - |
| 0.033 | 611 | 0.004 | 28.72 | -15.61 | -9.33 | R parahippocampal G | - |
|  |  |  | 26.9 | -11.7 | -10.79 | R amygdala | - |
|  |  |  | 19.53 | -7.76 | -12.34 | R amygdala | - |
|  |  |  | 39.93 | -2.1 | -13.26 | R sub-gyral | 21 |
|  |  |  | 28.85 | -11.01 | -17.9 | R parahippocampal G | - |
|  |  |  | 32.6 | 0.33 | -18.56 | R amygdala | - |
| **Hypometabolic areas at BS relative to CTR** | | | | | | | |
| 0 | 8740 | 0 | -27.4 | 13.17 | 44.7 | L Middle Frontal G | 6 |
|  |  |  | 33.58 | 4.88 | 50.35 | R Middle Frontal G | 6 |
|  |  |  | 30.02 | 22.36 | 44.74 | R Middle Frontal G | 8 |
|  |  |  | 32.22 | 39.52 | 22.98 | R Middle Frontal G | 10 |
|  |  |  | 13.31 | 20.23 | 47.86 | R Cingulate G | 32 |
|  |  |  | -34.99 | -4.61 | 53.7 | L Middle Frontal G | 6 |
|  |  |  | 43.04 | 2.67 | 34.09 | R Precentral G | 6 |
|  |  |  | -44.04 | 4.29 | 39.97 | L Middle Frontal G | 6 |
|  |  |  | 44.87 | 11.63 | 38.57 | R Middle Frontal G | 9 |
|  |  |  | -36.44 | 25.79 | 31.33 | L Middle Frontal G | 9 |
|  |  |  | 47.09 | 12.54 | 9.87 | R Precentral G | 44 |
|  |  |  | 38.06 | 28.89 | -3.15 | R Inferior Frontal G | 47 |
|  |  |  | -43.88 | 3.64 | 27.3 | L Inferior Frontal G | 9 |
|  |  |  | -16.25 | 33.6 | 46.82 | L Superior Frontal G | 8 |
|  |  |  | 45.26 | 29.15 | 13.21 | R Inferior Frontal G | 46 |
| 0 | 1668 | 0 | -31.3 | -70.48 | 34.91 | L Precuneus | 19 |
|  |  |  | -47.84 | -60.38 | 28.38 | L Superior Temporal G | 39 |
| 0 | 1689 | 0 | 33.49 | -68.96 | 36.15 | R Precuneus | 19 |
|  |  |  | 46.64 | -62.04 | 22.61 | R Middle Temporal G | 39 |
| 0.013 | 537 | 0.002 | -47.3 | -58.24 | -12.85 | L Fusiform G | 37 |
|  |  |  | -50.89 | -40.93 | -16.68 | L Inferior Temporal G | 37 |
|  |  |  | -56.5 | -37.7 | -11.06 | L Middle Temporal G | 20 |
|  |  |  | -60.2 | -26.67 | -8.28 | L Middle Temporal G | 21 |
|  |  |  | -56.34 | -21.92 | -18.57 | L Inferior Temporal G | 20 |
| 0 | 1859 | 0 | -5.23 | -63.98 | 25.15 | L Precuneus | 31 |
|  |  |  | 5.89 | -56.69 | 26.04 | R Cingulate G | 31 |
|  |  |  | 5.78 | -46.45 | 37.81 | R Precuneus | 31 |
|  |  |  | -3.44 | -42.51 | 36.23 | L Cingulate G | 31 |
|  |  |  | -5.25 | -34.87 | 35.12 | L Cingulate G | 31 |

Peak coordinates and cortical regions in each cluster are ordered downward from the highest Z-score peak. Abbreviations: BS, baseline; CTR, control group; BA, Brodmann area; R, right; L, left; G, gyrus

**Supplementary Table 4s. Correlation analysis between VOIs and clinical scales, at baseline (BS) and follow-up (FU)**

*Statistically significant (p < 0.05)

**Supplementary Table 5s. Backwards regression models**

| Predictors | Estimate | p-value | SE | VIF | *R*^2^ | AIC | BIC |
| --- | --- | --- | --- | --- | --- | --- | --- |
| **Backwards generalized linear model for CASE FU>2** | | | | | | | |
| mRS BS | 0.375 | 0.150 | 0.25 | 1.16 | 0.38 | 78.9 | 84.3 |
| VOI-B1 BS | -6.58 | **0.026*** | 2.72 | 1.18 |  |  |  |
| VOI-A BS | -3.11 | 0.287 | 2.83 | 1.13 |  |  |  |
| **Backwards generalized linear model for mRS FU>2** | | | | | | | |
| CASE BS | -0.26 | 0.139 | 0.17 | 2.64 | 0.208 | 79.3 | 84.8 |
| mRS BS | -2.11 | **0.049*** | 2.80 | 1.08 |  |  |  |
| VOI-A BS | 0.80 | 0.460 | 0.38 | 2.66 |  |  |  |
| **Backwards binomial linear model for relapse (no=0; yes=1)** | | | | | | | |
| VOI-B1 BS | -20.97 | **0.032*** | 9.77 | 2.55 | 0.275 | 27.6 | 30.8 |
| VOI-B2 BS | 7.56 | 0.150 | 5.25 | 2.55 |  |  |  |
| **Backwards binomial linear regression for therapy (1°line=0; 1°+2°line therapy=1)** | | | | | | | |
| Age | -0.11 | **0.020*** | 0.046 | 1.15 | 0.45 | 22.9 | 26.2 |
| Sex | -2.18 | 0.098 | 1.131 | 1.15 |  |  |  |

*Statistically significant (p < 0.05)

**Supplementary Figure 1s – ROC analysis**

**
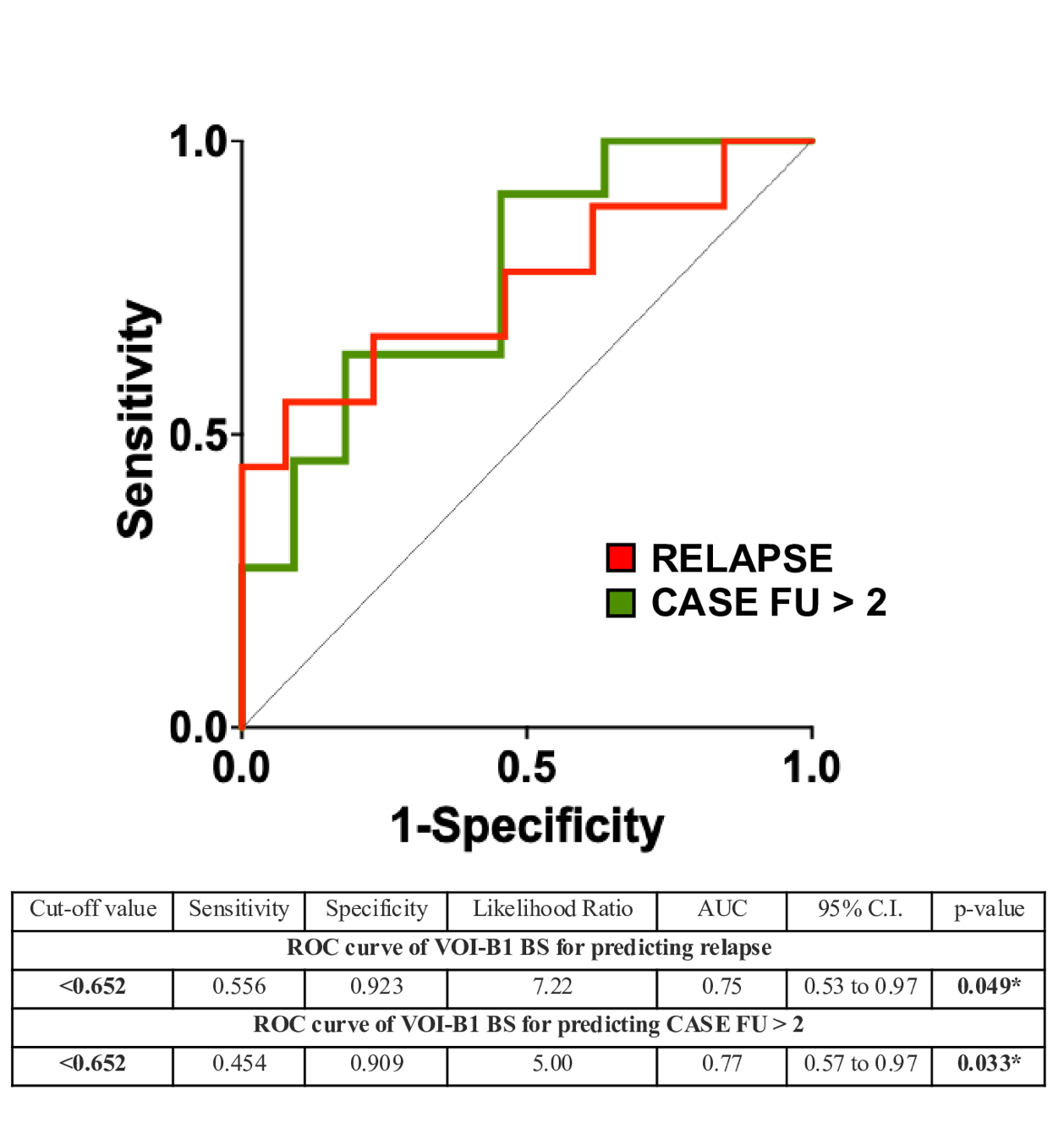
**

**Supplementary Figure 1s legend.** Receiver operating characteristic (ROC) curves for baseline VOI‑B1 in predicting (red) relapse and (green) a CASE score > 2 at follow‑up. The optimal threshold of 0.652—selected on the basis of the highest likelihood ratio—serves as the best discriminative cut‑off for both outcomes.

*Statistically significant (p<0.05) Abbreviations: ROC, Receiver Operator Characteristic; AUC, Area under the ROC curve; C.I., confidence interval; CASE, Clinical Assessment Scale in Autoimmune Encephalitis; BS, baseline; FU, follow-up
