# Supplementary material for: Longitudinal changes in [18F]FDG PET brain metabolism as a prognostic marker in autoimmune encephalitis
Source: Eur J Nucl Med Mol Imaging. 2025 Aug 25;53(2):1183–97. doi: 10.1007/s00259-025-07526-2 (PMC12830435; doi:10.1007/s00259-025-07526-2)
Supplement: Supplementary file 3 — (DOCX 1.53 MB) [file 259_2025_7526_MOESM3_ESM.docx]

**Longitudinal changes in [^18^F]FDG PET brain metabolism as a prognostic marker in autoimmune encephalitis**

Denise Cerne^1^, Stefano Raffa^2^, Giulia Benvenuto^1^, Giacomo Rebella^2^, Pietro Mattioli^1,2^, Giacomo Bavestrello^1^, Anastasia Lechiara^3^, Emanuela Maria Mobilia^3^, Dario Arnaldi^1^**^,^**^2^, Flavio Villani^2^, Luca Roccatagliata^2,4^, Giampaola Pesce^3^, Matteo Pardini^1,2^, Silvia Morbelli^5,^⁶, Antonio Uccelli^1,2^, Luana Benedetti^2^, Federico Massa^1,2^

^1^ Department of Neuroscience, Rehabilitation, Ophthalmology, Genetics, Maternal and Child Health, University of Genoa, Genoa, Italy

^2^ IRCCS Ospedale Policlinico San Martino, Genoa, Italy.

^3^ Autoimmunology Laboratory, IRCCS Ospedale Policlinico San Martino, Genoa, Italy

^4^ Department of Health Sciences (DISSAL), University of Genoa, Genoa, Italy

^5^Department of Medical Sciences, University of Turin, Italy

⁶ Nuclear Medicine Unit, Azienda Ospedaliero-Universitaria Città della Salute e della Scienza di Torino, Turin, Italy.

Corresponding author

Federico Massa, MD

Department of Neuroscience, Rehabilitation, Ophthalmology, Genetics, Maternal and Child Health (DINOGMI), University of Genoa, Largo Daneo 3, 16132 Genoa, Italy

e-mail: fedemassa88@gmail.com

**ABSTRACT**

**Purpose:** Recent advancements in autoimmune encephalitis (AE) have enhanced diagnosis and management, but predicting long-term outcomes remains challenging. This study aims to evaluate longitudinal changes in brain [^18^F]FDG PET patterns in AE patients to identify specific regional metabolic variations and predict clinical outcomes.

**Methods:** This longitudinal study compared brain [^18^F]FDG PET scans of 22 AE patients at baseline (BS) and after treatment follow-up (FU) using voxel-wise paired t-tests. Significant clusters with at least 100 voxels and p<0.05 were identified and designated as volumes of interest (VOIs). The VOI values were correlated with main clinical outcomes using partial Spearman’s tests, and their prognostic significance was assessed through regression models.

**Results:** Three VOIs showed significant metabolic changes between baseline (BS) and follow-up (FU) assessments. VOI-A, which was relatively hypermetabolic at BS, included the caudate-thalamus-parahippocampal region, right amygdala, and anterior cingulate cortex. VOI-B1 and VOI-B2, relatively hypometabolic at BS, corresponded to the right fusiform gyrus, precuneus, and temporo-parietal cortex, respectively. Poorer metabolic recovery in all VOIs to normalcy correlated with greater disability (mRS) in both acute and post-therapy phases. Lower metabolism in BS VOI-B1 predicted higher Clinical Assessment Scale in Autoimmune Encephalitis (CASE) score at FU and relapses, while lower age was a significant predictor of escalation to second-line treatment.

**Conclusions:** Longitudinal [^18^F]FDG PET reveals distinct regional metabolic changes paralleling clinical recovery post-treatment in AE. Temporo-parietal metabolism correlates with relapses, clinical severity, and functional impairment, highlighting [^18^F]FDG PET as a biological tracker of disease activity throughout AE and its prognostic value.  ~~potential role of metabolic imaging in guiding treatment and prognosis.~~

Key words: prognosis; autoimmune encephalitis; FDG-PET; biomarkers

# INTRODUCTION

In recent years, the knowledge and management of autoimmune encephalitis (AE) have significantly advanced, reflecting an increasing awareness of diverse clinical presentations and the development of more precise diagnostic tools. As a group of conditions characterized by immune-mediated brain inflammation brain, AE can present with a wide range of neurological and psychiatric symptoms, including seizures, cognitive decline, movement disorders, and behavioural changes [1–3]. Early and accurate diagnosis is crucial, as timely treatment can lead to substantial recovery in many patients [4].

In 2016, Graus et al.[5] proposed the currently used diagnostic criteria for AE. These criteria emphasized the importance of integrating various diagnostic modalities to achieve a reliable, timely diagnosis, prompting early treatment. Clinical symptoms serve as the foundation, but diagnostic accuracy improves significantly when they are combined with findings from imaging techniques such as brain magnetic resonance imaging (MRI), cerebrospinal fluid (CSF) analysis, and EEG findings that may reveal characteristic patterns of neural activity [5]. The diagnosis is confirmed by identification of specific neuronal antibodies, although seronegative AEs exist [5]. Despite significant advances in understanding its pathophysiology, clinical heterogeneity, and treatment, predicting long-term outcomes in AE remains challenging. This underscores the critical need for further research into prognostic factors, particularly the complex interplay among inflammation, neuronal/synaptic dysfunction, and recovery.

Currently, the Clinical Assessment Scale in Autoimmune Encephalitis (CASE) score [6] and the anti-NMDAR Encephalitis One-Year Functional Status (NEOS) score [7] are the sole tools available to physicians for assessing prognosis in AE. The CASE score predicts poor functional status (defined as a Modified Rankin Scale, mRS, greater than 2) one year after discharge across all types of AE, based on acute-phase clinical symptoms. It also serves as a guide for escalating to second-line treatment when first-line interventions are insufficient [8–10]. In contrast, the NEOS score is specifically tailored to anti-NMDAR encephalitis and incorporates five independent predictors of poor functional outcomes, namely cerebrospinal fluid (CSF) pleocytosis, lack of improvement one month after treatment, ICU admission, treatment delays over one month, and abnormal MRI findings. Despite their utility, both scores have limitations. The CASE score emphasizes clinical symptoms, potentially neglecting fluid or imaging information, while the NEOS score is limited to anti-NMDAR encephalitis and lacks generalizability.

Among other potential prognostic markers for AE, CSF cytokine profiles and EEG findings have attracted particular attention, although results across studies have been inconsistent. Several cytokines have been proposed as potential biomarkers of clinical and inflammatory activity as well as predictors of treatment response and outcome [11,12]. Nonetheless, no significant correlation with clinical outcome at one year has been established [13]. Regarding EEG, characteristic findings associated with different stages of disease and clinical severity have been described in anti-NMDAR-AE, but these lack specificity [12,14]. Furthermore, no consistent EEG patterns have been identified for other types of AE [15].

[^18^F]FDG PET has become increasingly important in diagnosing AE [16]. [^18^F]FDG PET offers functional imaging perspective that is especially valuable in early-stage or atypical cases, where MRI may fail to detect significant changes and differentiation from other neurological disorders is challenging. Therefore, over time the role of [^18^F]FDG PET in AE has evolved beyond merely being a secondary tool, as initially proposed by Graus et al., 2016 [5]. It has become an integral part of the diagnostic work-up, offering unique insights into the underlying neuroinflammation and metabolic alterations. Different brain metabolic patterns related to specific autoantibodies against intracellular and against surface antigens have been thoroughly described, highlighting the value of [^18^F]FDG PET in differentiating AE subtypes [17–21]. Furthermore, these [^18^F]FDG PET findings are associated with clinical symptoms, disease severity, and recovery after therapy [5,22–24]. The progressive normalization of brain [^18^F]FDG PET emphasizes the ability to monitor disease activity suggesting its potential role as a prognostic tool in AE. However, only a few studies have explored this field with varying yet encouraging results. For instance, Liu et al. [25] observed that an increased standardized uptake value (SUV) in the medial temporal lobe structures, including the hippocampus and amygdala, may serve as a prognostic marker in seropositive AE being correlated with higher mRS score following treatment. In contrast, Dai et al. [26] described that hypometabolism in the right superior frontal gyrus, coupled with hypermetabolism in the brainstem, is associated with poor outcome. However, neither study included re-examination with [^18^F]FDG PET after the acute phase, limiting the ability to monitor dynamic brain metabolic changes. This may be crucial for tracking disease course, yet there is no definite evidence supporting the clinical value of re-assessment compared to baseline.

Building on these foundations, our study aimed to evaluate the longitudinal changes in brain [^18^F]FDG PET before and after treatment in a heterogeneous group of AE patients. We analysed its practical utility to identify specific brain regional patterns of metabolic variation over time, correlate these changes with recovery and predict clinical outcomes, including relapses and the need for escalation from first- to second-line treatments. By addressing these questions, our research aims to contribute to a more nuanced understanding of the role of [^18^F]FDG PET in the management of AE patients, regardless of the antibody subtype, ultimately enhancing personalized treatment strategies and improving patient outcomes.

**METHODS AND MATERIALS**

**Study population**. A total of 22 AE patients were included consecutively from IRCCS Policlinico San Martino (Genoa, Italy) and Sant’Andrea (La Spezia, Italy) hospitals between January 2013 and December 2023, from an in-house dataset of 45 patients diagnosed with AE in the same timeframe. As for the inclusion criteria, they fulfilled the clinical diagnostic criteria for *“definitive autoimmune limbic encephalitis”,* *“definite anti-NMDA receptor encephalitis”,* or *“autoantibody-negative but probable autoimmune encephalitis”* (Graus et al, 2016); they performed brain [^18^F]FDG PET both at the time of AE diagnosis (baseline timepoint, BS) and after immunomodulatory therapy, at least 3-6 months after the previous one (follow-up timepoint, FU). The average time between performing the BS and FU [^18^F]FDG PET was 11.0 months. Patients were clinically observed every six months for a mean of 4 years, ranging from 5 to 156 months.

The following data were collected for each patient: sex and age at onset of AE, presence and type of autoantibodies, main presenting symptoms, i.e., cognitive impairment, epilepsy, psychiatric disorders, and others (e.g., speech disorders, diffuse pain, asthenia and profuse sweating), CASE and mRS score at BS and FU timepoints, as well as relapse occurrence and treatments considered as 1^st^-line (high-dose steroid, plasmapheresis, or intravenous immunoglobulins, IVIG) or 2^nd^-line (Rituximab or other immunosuppressants, such as azathioprine) during the overall clinical observation.

**Autoantibodies detection**. Neuronal surface antibodies were tested on serum and or CSF samples through indirect immunofluorescence (IFI) on transfected EU 90 cells (Euroimmun, Lubeck, Germany), following manufacturer's instructions and recommended dilutions. Sera were tested for anti-NMDAr, -AMPAr1, -AMPAr2, -GABArB1/B2 -DPPX, -CASPR2, -LGI1 total IgG antibodies at dilution of 1:10. CSF samples were tested undiluted. Intracellular neuronal antibodies were tested through IFI on primate cerebellum (Euroimmun, Lubeck, Germany) and with line-blot immunoassay (Ravo Diagnostika, GmbH) for the detection of Hu, Yo, Ri, CV2, Amphiphysin, Ma1, Ma2, SOX1, DNEr, Zic4, GAD65, PKCγ, Recoverin and Titin following manufacturer's instructions.

## [^18^F]FDG PET protocol and image pre-processing. We followed European Association of Nuclear Medicine guidelines for PET/CT in effect at the time of scan acquisition [27]. The PET/CT scanner was a Siemens Biograph 16. All patients were asked to fast for at least 6 h, and fasting blood glucose levels could not exceed 8 mmol/L. The injection dose was 185-250 MBq, and the imaging agent was 18F-FDG. After injection, the patients were required to rest quietly and were isolated in a dedicated room to ensure minimal auditory and visual stimulation. The brain imaging acquisition time was 45 min after injection. For imaging reconstruction ordered subset-expectation maximization (OSEM) was used, while MATLAB and Statistical Parametric Mapping software (SPM12, Wellcome Trust Centre for Neuroimaging, London, United Kingdom) were used for further processing. The PET images were normalized into a specific FDG-PET template in the MNI stereotaxic space [28] and resampled to 2×2×2 mm^3^ voxels, then spatially smoothed using a 10-mm isotropic Gaussian filter.

## [^18^F]FDG PET analysis

*Metabolism changes at BS relative to normalcy.* After pre-processing, the smoothed images were subjected to a voxel-based analysis (VBA) of the whole brain comparing the [^18^F]FDG PET in AE at acute phase (BS) and a control (CTR) group by two-sample t-test with age as a covariate.

The CTR group was composed of 30 ~~healthy~~ individuals without objective neurocognitive disorders - matched for age and sex with the AE patients (15 females; age 57.5±20.1, range 15.5-84.7 years) - who underwent [^18^F]FDG PET within the framework of previous research as healthy controls (n=25) or in the diagnostic workup for other systemic disorders (n=5) without evidence of central nervous system alteration at the time of the scan. Their health status at the time of the [^18^F]FDG PET scan was verified through medical records and their scans were judged to be normal by expert readers.

*Longitudinal metabolism changes from BS to FU.* Images were subjected to VBA of the whole brain comparing in the same patients the scans at BS and FU timepoints (paired t-test). In a first analysis, voxels with a significantly different metabolism value in the two groups regardless of direction (F-contrast) were identified. This preliminary, exploratory, step was deemed essential, as it allowed us to account for the potential coexistence of hyper- and hypometabolic regions within the same patient as an expression of the disease. Then, considering *a priori* FU as the normalization of the disease overtime, a T-contrast was applied to test the direction of these differences, i.e., relatively hypometabolic (-1; 1) or hypermetabolic (1; -1) regions (hereafter volumes of interest, VOIs) at BS relatively to FU.

In all VBAs we normalized the raw metabolic values to whole-brain activity. This adjustment accounts for the potential involvement of cortical and subcortical structures in the encephalitic process, preventing the identification of any unaffected reference region *a priori* serving as a reliable reference. To mitigate the risk of false findings caused by relative hypermetabolic areas as an artifact of global mean normalization in SPM, the "gray matter threshold" was raised from the default value of 0.8 to 1. This adjustment excluded voxels from hypometabolic regions as well, following the approach used by other researchers when studying neurological conditions that may involve both hypometabolism and hypermetabolism [29,30]. Drawing on experience from neurodegenerative research, standard proportional global mean scaling was applied to each image under the assumption that local changes do not significantly affect the global mean glucose uptake, thus preventing underestimation of hypometabolism and overestimation of hypermetabolism in SPM-based statistical analyses [31,32]. The result of the analyses was an SPM t-Map, indicating clusters of statistical significance. We applied a statistical threshold of p<0.001 at the voxel-level and explored additional more permissive thresholds up to p=0.005 to balance between type I and type II errors [33] as used in previous [^18^F]FDG PET studies by our group [34,35] in consideration of the relatively low sensitivity of PET analysis in repeated measures. A stricter voxel-level threshold would be excessively conservative due to the inherent signal-to-noise characteristics of [¹⁸F]FDG PET imaging, particularly in small cohorts. However, we were rigorous in considering only clusters containing at least 100 voxels and that reached statistical significance (p<0.05) for multiple comparisons at the cluster level after correction for family-wise-error (FWE), thus minimizing the probability of spurious findings. This approach is consistent with previous PET studies and established guidelines for automated evaluation of [¹⁸F]FDG PET scans [36].

Cluster coordinates were converted using Ginger Ale and Talairach Client software for mapping onto the Brodmann-classified Talairach 3D atlas. Using the Marsbar (MARSeille Boîte À Région d’Intérêt) toolbox implemented in SPM12, we calculated the average count density of the distinct relatively hypermetabolic or hypometabolic VOIs, scaling them against whole-brain counts as a reference. The VOI metabolic values of AE (at BS and FU) and CTR were then used in the subsequent VOI-based statistical analyses.

**Statistical analysis**

**Demographic, clinical and treatment data***.* Clinical-demographic data with relapse occurrence and treatments were described in patients in terms of mean, standard deviation (continuous variables) and percentages (categorical variables). Continuous variables were tested to determine whether they conformed to a normal distribution by the Shapiro–Wilk normality test. CASE and mRS scores at BS and FU were compared in patients by paired two-tailed T-Test or Wilcoxon rank sum test according to normal distribution of the values. Age, sex, CASE and mRS scores were also compared in patients with or without clinical relapse and in patients treated with 1^st^-line therapy compared to those escalating to 2^nd^-line treatments during the entire clinical observation by unpaired two-tailed T-Test or Wilcoxon rank sum or Chi-square test χ² when appropriate. VOI-based analysis*.* We assessed whole‑brain‑scaled metabolic count density within each VOI for the AE (at BS and FU) and CTR groups using a one‑way ANOVA with Bonferroni‑corrected post‑hoc comparisons. This analysis aimed to establish whether each VOI was relatively hypo‑ or hypermetabolic at BS versus FU, with metabolic normalization after therapy inferred when FU values overlapped those of the CTR group. VOI values were also compared in the AE patients after stratification according to clinical outcomes, including dichotomized CASE and mRS scores (based on median cut-offs), relapse occurrence (yes/no), and treatment type (1^st^-line/1^st^+2^nd^-line). Clinical scales (i.e., CASE and mRS) were correlated with the distinct VOI values at BS or FU by partial age-adjusted Spearman’s test. The metabolic values of the BS VOIs were then used in regression models to test their prognostic role using the previous clinical variables as separate outcomes. Generalized linear model (GLM) was used for continuous outcome variables (CASE FU and mRS FU), while binomial logistic model (BLM) was used for dichotomic outcomes, namely relapse occurrence (yes/no) and treatment type (1^st^-line/1^st^+2^nd^-line). Multicollinearity among covariates was assessed using Spearman's correlation and variance inflation factor (VIF), with a 2.5 cutoff commonly used in the literature [37]. AIC and BIC were used to select the most appropriate regression model through a backward elimination of variables approach, starting with a full model with the main clinical variables at baseline (age, sex, CASE BS, mRS BS) and VOI BS values. The model minimizing both AIC and BIC criteria was preferred, balancing fit and complexity. ROC analyses were performed using the strongest predictor identified by our linear models against the corresponding outcomes. For each analysis, sensitivity was plotted against 1 – specificity, the area under the curve (AUC) was calculated via the nonparametric DeLong method, and the optimal cut‑off was defined as the point maximizing the positive likelihood ratio.

## A p‐value < 0.05 was considered significant in all the analysis which were performed with Jamovi software (version 2.3) and GraphPad Prism 10 (v10.4.2).

#

# RESULTS

# Demographic, clinical and treatment data. Among the 22 patients with AE, we classified 11 patients (50%) with definite limbic AE, 4 (18,2%) with definite NMDAR-AE, and 7 (31,8%) with probable seronegative AE, according to Graus criteria [5]. Clinical relapses occurred in 9 patients (41%). All patients were treated with 1^st^-line therapy, either single (36.4%) or combined (63.6%). Additionally, 10 out of 22 patients (45.5%) underwent 2^nd^-line therapy. As for demographic values, age significantly differed in patients escalating to 2^nd^-line treatment (p=0.007) but not according on relapse occurrence (p=0.058) (Table 1).

# CASE and mRS scores are presented in Table 1 for all patient groups and in the Supplementary Table 1s for each AE subtype. As expected, at FU there was a significant improvement in both CASE (4.4±2.75 at BS vs 2.5±1.5 at FU, p=0.003) and mRS (3.1±1.2 at BS vs 2.0±1.35 at FU, p=0.002) in the entire cohort. Notably, although all subjects showed moderate to severe disability in the acute phase, the anti-NMDAR AE patients had greater clinical severity (i.e., higher CASE score) at BS. At FU, only those patients with onconeural antibody-associated AE showed an unfavorable outcome (i.e., mRS>2), likely driven by the underlying malignancy.

## [^18^F]FDG PET analysis

*Metabolism changes at BS relative to normalcy.* Compared with controls, AE patients at BS exhibited relative hypermetabolism in bilateral cerebellum and right hippocampus–amygdala complex. Conversely, they showed widespread cortical hypo-metabolism, most pronounced in the bilateral frontotemporal and the precuneus/posterior cingulate regions. These findings are shown in **Figure 1,** whereas detailed peak coordinates, significance levels, and corresponding Brodmann areas are listed in **Supplementary Table 2s.**

## *Longitudinal metabolism changes from BS to FU*

## **F-contrast BS vs FU.** As detailed in Table 2, we found two distinct VOIs significantly differing in terms of metabolism in paired scans at BS relative to FU: i) VOI-A formed by the bilateral thalamus and caudate nucleus, the right parahippocampal and anterior cingulate cortex, and the right amygdala, and ii) VOI-B encompassing the right temporo-parietal cortex and precuneus.

T-contrast BS vs FU. When predefining the direction of regional differences, we assessed that VOI-A was relatively hypermetabolic at BS compared to FU (contrast vector of 1; -1). Conversely, VOI-B included relatively hypometabolic regions (contrast vector of -1; 1) and could be further divided into two subclusters, namely VOI-B1 and VOI-B2, both involving the right temporo-parietal regions. Specifically, VOI-B1 corresponds to the fusiform gyrus and the middle and inferior temporal gyri, while VOI-B2 involves the precuneus, superior and middle temporo-occipital and supramarginal giri, and inferior parietal lobule in the right hemisphere.

Table 3 provides details on cluster extent and statistical significance, coordinates, and corresponding Brodmann areas for the above mentioned VOIs, which are displayed in Figure 2.

*VOI-based analyses.* The whole-brain-scaled metabolic count density values of the distinct VOIs significantly differed in the group comparisons (BS, FU, CTR) (p<0.001 for all VOIs). Post-hoc analysis revealed higher VOI-A alongside lower VOI-B1 and VOI-B2 values in BS with respect to both FU (VOI-A p<0.001, VOI-B1 p<0.001, VOI-B2 p=0.002) and CTR (VOI-A p=0.008, VOI-B1 p=0.013, VOI-B2 p=0.044). The absence of a significant difference between VOI values of patients at FU and those of the CTR group supported a trend toward metabolic normalization after treatment (Figure 3, Supplementary Table 3s).

The whole-brain-scaled metabolic count density values of the distinct VOIs also significantly differed in AE patients grouped according to dichotomized clinical outcomes (Figure 3, Supplementary Table 3s). Patients with CASE BS > 3 and CASE FU > 2 had lower VOI-B2 BS (p=0.031) and VOI-B1 BS (p=0.031), respectively, than those patients with CASE BS ≤ 3 and CASE FU ≤ 2. Those with mRS BS >2 exhibited higher VOI-A FU (p=0.018) alongside lower VOI-B1 FU (p=0.035) and VOI-B2 FU (p=0.026) values than those patients with mRS BS ≤ 2. Similarly, patients with mRS FU>2 had lower VOI-B1 FU (p=0.021) and VOI-B2 FU (p=0.009) values than those patients with mRS FU ≤ 2. Notably, patients who relapsed clinically had lower VOI-B1 BS (p=0.020). No significant differences were found in VOI values according to the CASE BS score or line of treatment.

In the correlation analysis, CASE FU was negatively correlated to VOI-B1 BS (r=-0.53, p=0.014), while mRS BS scores were negatively correlated with VOI-B1 FU and VOI-B2 FU (r=-0.50, p=0.021 and r=-0.44, p=0.043, respectively). Noteworthy, no correlations were found in terms of the VOI values according to the CASE BS score neither to the mRS FU scores. All results of the correlation analysis coherently reflected the VOI comparisons based on dichotomized clinical variables, except for the CASE BS and mRS FU where we observed only a trend toward statistical significance (Supplementary Table 4s).

In the GLM analysis of prediction using BS variables, the best model to predict the CASE FU included mRS BS, VOI-A BS, and VOI-B1 BS. Notably, VOI-B1 BS was the most significant predictor in the model (p=0.026, AIC=78.9, BIC=84.3, R²=0.38). Regarding the long-term disability, the best model to predict mRS FU considered three variables (CASE BS, VOI-A BS, mRS BS), showing that mRS BS was the only predictor (p=0.049, AIC=79.3, BIC=84.8, R²=0.208).

In the BLM analysis of prediction using BS variables, the best model to predict relapse occurrence included two BS variables (VOI-B1 BS, and VOI-B2 BS), with VOI-B1 BS being again the best predictor (p=0.032, AIC=27.6, BIC=30.8, R²=0.275). As for the need for escalation therapy, the best BLM model included two variables at BS (age, sex ~~VOI-A BS~~), with age being a significant predictor (p=0.020, AIC=22.9, BIC=26.2, R²=0.45 ~~p=0.014, AIC=25.5, BIC=28.8, R²=0.36~~). The results of regression models are detailed in Supplementary Table 5s.

VOI‑B1 BS emerged from the linear models as the strongest predictor of both relapse and clinically significant CASE scores at follow‑up. ROC analysis (displayed in Supplementary Figure 1s) yielded AUCs of 0.75 for relapse and 0.77 for CASE > 2, reflecting good discriminative power. By selecting the VOI‑B1 BS cut‑off that maximized the positive likelihood ratio, we identified a single threshold of 0.652 (whole‑brain‑scaled count density), which provided balanced sensitivity (0.56 for relapse; 0.45 for CASE > 2) and specificity (0.92 and 0.91, respectively).

**DISCUSSION**

In this study, we investigated the prognostic value of brain metabolism by analysing its longitudinal changes during the acute phase and following immunomodulatory therapy in a heterogeneous group of patients with autoimmune encephalitis (AE).

Using a voxel-based approach with paired [^18^F]FDG PET scans obtained at BS and FU timepoints, we observed specific longitudinal regional changes. Certain regions exhibited relative hypermetabolism while others were hypometabolic at BS, under the assumption of normalization after therapy - which was confirmed by the similarity between the metabolic values of these regions at FU and those extracted from the CTR. This normalization corresponded to clinical improvements, as verified by both CASE and mRS scores at FU. Precisely, we identified areas of relative hypermetabolism in the caudate-thalamus-parahippocampal region, right amygdala and anterior cingulate cortex (i.e., VOI-A), alongside regions of relative hypometabolism in the right temporo-parietal cortex and precuneus (i.e., VOI-B1 and B2). This pattern of metabolic changes defines a distinctive acute-phase profile, which gradually resolves with treatment, highlighting the dynamic nature of metabolic alterations. Interestingly, larger metabolic alterations– either in the hypo- or hypermetabolic direction – emerged in BS compared to CTR. Precisely, many cortical areas – including prominently frontal-temporal and precuneus/posterior cingulate regions – were relatively hypometabolic, consistently with widespread hypometabolism patterns described in AE [19]. Conversely, the right hippocampus and cerebellum were relatively hypermetabolic. Albeit cerebellar hypermetabolism is described in AE [38,39], this signal is probably an artifact caused by the whole-brain proportional scaling. When diffuse cortical hypometabolism lowers the global mean, spared areas—like the cerebellum— can appear hypermetabolic even when absolute metabolism is unchanged. This persisted despite our preventive attempts to curb the normalization and scaling bias. Visual review **confirmed that no genuine cerebellar hypermetabolism was present in our AE cohort.**

Our findings highlight the added role of longitudinal [^18^F]FDG PET scan assessment to specifically uncover those critical brain regions where metabolism shifts dynamically over the disease course and parallel recovery—regions that do not necessarily coincide with all those found abnormal at BS relative to normalcy. The hypermetabolic VOI-A lies within the limbic system - including the hippocampus, amygdala, and cingulate cortex - a network uniquely prone to autoimmune inflammation. Neurons within limbic structures express higher neuronal synaptic activity and metabolic demands alongside elevated levels of antigens such as NMDA, GABA, LGI1, and CASPR2 receptors, which are frequent targets of AE-associated autoantibodies [40]. Exposure to autoantibodies shifts the excitation-inhibition balance of synaptic inputs towards hyperexcitation[41]. Moreover, the regional blood-brain barrier exhibits increased permeability, facilitating the infiltration of circulating autoantibodies and immune cells, while a dense array of cytokine/chemokine receptors further amplifies local inflammatory signaling [42]. These processes drive clinical manifestations of AE, such as memory impairment, mood and motor disturbances, and seizures, and are reflected in [^18^F]FDG PET as relative hypermetabolism due to heightened glucose demand and consumption [5,19].

In AE, hypometabolism results from autoantibody interactions with target receptors, causing inhibition, internalization, or ion channel blockade. These mechanisms reduce receptor density, impair function, lower neuronal activity, and disrupt neural connectivity [18,19]. Consequently, cortical hypometabolism likely reflects functional impairments propagated through cortical and subcortical networks. Focusing on the areas of relative hypometabolism in the longitudinal assessment, VOI-B1 includes the right fusiform, middle and inferior temporal gyri, while VOI-B2 involves the precuneus, supramarginal, superior and middle temporo-occipital gyri, and inferior parietal lobule in the right hemisphere. These regions are closely interconnected forming part of complex cortico-subcortical (e.g., the limbic system) and cortico-cortical circuits, and are critically involved in a range of cognitive and behavioural functions. Specifically, the fusiform gyrus relates to the hippocampus, inferior and superior temporal gyrus, occipital regions, supramarginal gyrus, and the default mode network (DMN) [43]. Functionally, it is involved in emotion perception, facial recognition, language processing, and semantic cognition [44,45]. Similarly, the middle temporal gyrus is implicated in lexical comprehension and semantic cognition [46,47] and belongs to the ventral visual stream [48]. The precuneus is a key node of the DMN that is primarily active during cognitive leisure and self-monitoring processes [49,50]. Alterations in the DMN have been described in temporal lobe epilepsy, mood disorders, and Alzheimer's disease [51–54]. The action of the DMN is opposed to that of the frontoparietal network (FPN), which peaks during task involvement and cognitive effort. The dynamic “switch” between these two networks depends on the activity of the salient network (SN), whose core driver is represented by the right amygdala [55]. Structural and functional hemispheric asymmetries, driven by connectivity differences from lateralization and pathology-related reorganization, are well-documented in the literature [56–58]. Our finding of prominent involvement of the right hemisphere are in line with previous studies that emphasize the importance of these regions in cognitive and psychiatric symptoms [44,53,59,60].

This network disruption accounts for behavioural and cognitive - especially executive and attentional - deficits observed in AE [61,62]. In anti-LGI1-AE, inflammation and damage in the MTL, hippocampus and amygdala disrupt functional connectivity within large-scale networks – including DMN, FPN, and SN – and cause typical cognitive and neuropsychiatric disorders [63]. Similarly, in anti-NMDAR-AE hippocampal decoupling from the DMN and abnormal brain glucose metabolism - characterized by a frontal-to-occipital gradient - mirror functional connectivity alterations, such as those in the sensorimotor visual and ventral attention networks [63,64].

The coexistence of relative hyper- and hypometabolism in AE is consistent with prior evidence [18,20,25,26,63,65]. This dual metabolic signature underscores AE heterogeneity, where foci of neuroinflammation or excitotoxicity concur with areas of decreased activity from synaptic failure. Antibody class modulates the balance and influences the predominant metabolic pattern, with a shift either towards hyper- or hypometabolism relative to normal levels. Regional, mainly limbic, hypermetabolism predominates with intracellular onconeural antibodies driven by T-cell-mediated inflammation, whereas hypometabolism is typical of subtypes with neuronal-surface antigen antibodies (NSAb), that mainly disrupt synaptic function [18,19]. Our cohort included NSAb-positive, intracellular-antibody, and seronegative cases, revealing a shared longitudinal metabolic trajectory among these subtypes from acute to post-treatment phases regardless of antibody profile. Metabolic count densities were extracted from VOIs at BS and FU timepoints to explore associations with clinical severity and disability (CASE and mRS scores), relapse occurrence, and the need for escalation to second-line therapy – key factors in patient management. In the acute BS phase, patients with higher relative hypometabolism within VOI-B1, covering mostly encompassing temporal regions - were more prone to relapse and greater FU clinical severity (i.e. CASE score>2). This suggests that metabolic dysfunction in these temporal areas, part of the DMN, predisposes patients to lasting neuronal and functional damage. This is a novel finding and emphasizes the prognostic value of hypometabolism. Notably, the median FU CASE fell in the mild range [10]., yet metabolic abnormalities still mirrored meaningful impairment, underscoring the sensitivity of [^18^F]FDG-PET beyond the limits of clinical presentation. In our cohort, a baseline VOI‑B1 BS whole‑brain‑scaled metabolic count density cut‑off of 0.652—corresponding to VOI‑B1 uptake below 65.2 % of mean whole‑brain activity—identified patients at highest risk of both relapse and elevated follow‑up CASE scores. By selecting this value to maximize specificity, we minimize false positives and ensure that individuals flagged as high risk truly warrant intensified monitoring or early intervention. If validated against normative data in larger, heterogeneous AE cohorts, this threshold could be directly translated into clinical practice to guide personalized patient management.

No BS VOI metric predicted poor long-term function (mRS > 2). Divergence from prior reports likely stems from analytical differences in [^18^F]FDG PET imaging analysis and patient cohort. Liu et al. [25] employed a visual SUV grading by manual drawing the region of interest (ROI) in only seropositive AE patients, while the VBA approach of Dai et al. [26] used smaller clusters (about 20 voxels) raising false-positive risk. Moreover, a visual grading might be more likely to identify hypermetabolism, compared with hypometabolism. whereas our voxel‑based analysis specifically highlighted regions of relative hypometabolism in a mixed cohort of seropositive and seronegative patients.

Escalation to second-line therapy was predicted solely by lower age in regression model with BS variables. Younger patients, more often harboring anti-NMDAR-AE and facing higher severity and relapse risk, tended to receive aggressive escalation. Thus, age—together with fewer comorbidities—guided clinicians in our cohort more than baseline metabolic patterns or CASE scores, while also accounting for comorbidities which may limit treatment escalation in older patients [66,67].

The originality of this work lies in linking the VOI metabolic values at FU in relation to clinical outcomes. Patients with greater functional disability in the acute phase (i.e., mRS BS > 2) exhibited persistent relative hypermetabolism in VOI-A and hypometabolism in VOI-B1 and VOI-B2 at FU in the comparison and correlation analyses. Persistently reduced metabolism in VOI-B1 and B2 at FU was associated with poorer long-term outcome (i.e. mRS FU>2). The affected regions, i.e, precuneus and right temporo-parietal-occipital cortices, is consistent with multidimensional cognitive deficits and psychiatric symptoms, as well as accumulating disability [68,69]. Thus, temporo-parietal dysmetabolism not only predicts relapses and acute severity but also mirrors a continuing pathological process that may prompt more aggressive interventions. This highlights [^18^F]FDG PET as a biological tracker of disease activity throughout AE.

A strength of this study is the longitudinal design. Each patient served as their own reference, enabling rigorous, VOI-based quantification of hyper- and hypometabolic shifts in a heterogeneous seropositive/seronegative cohort. Future studies in larger, more diverse cohorts could provide a more detailed and nuanced understanding of the specific role of each antibody subtype in these dynamic metabolic changes. Indeed, limitations include the small sample, which is inevitable in a rare disease, although it is similar to that of previous studies on this topic. Specifically, Dai et al. described 30 patients with probable/definite AE and Liu et al. reported 24 AE patients out of 32 evaluated by [^18^F]FDG PET [25,26]. The absence of formal neuropsychological batteries for AE [70,71] represents another limitation, largely due to the challenge of evaluating cognition in the acute phase—when altered consciousness and impaired sustained attention are common. However, because long‑term clinical deficits are often driven by cognitive and behavioural disturbances, future investigations should incorporate comprehensive, prospective neuropsychological assessments to elucidate correlations with distinct hypometabolic patterns. We also lacked serial EEG, precluding assessment of aberrant electrical activity contribution to hypermetabolism [72,73]. Finally, preliminary findings indicating tight coupling between [^18^F]FDG PET metabolism and perfusion encourage replication with MRI techniques, which avoid radiation and are more widely available [74].

In conclusion, this study underscores the value of assessing [^18^F]FDG PET in both the acute phase and post-treatment follow-up of AE patients. Acute metabolic alterations, particularly temporal and parietal hypometabolism, reliably signals relapse risk and greater clinical severity.

# Within this context, accurate [^18^F]FDG PET interpretation is paramount. Qualitative readings—especially by non-experts —may be skewed by reference scales that exalt acute hypermetabolism while overlooking hypometabolic changes. Semi-quantitative tools - now standard in major centers and widely adopted in neurodegenerative disorders [75,76], provide an objective voxel-wise reference ensuring hypometabolic changes are captured and management decisions are better informed. Future prospective studies should include evaluation of a larger population, encompassing all AE subtypes, to validate these findings and ~~pair~~ combine longitudinal[^18^F]FDG PET with comprehensive neuropsychological testing to clarify how specific metabolic patterns relate to cognitive outcomes and to refine personalised patient care.

**Statements and Declarations**

**Funding.** This work was partially supported by FRA2022/DINOGMI – Department of Neuroscience, Rehabilitation, Ophthalmology, Genetics, Maternal and Child Health, University of Genoa. F Massa was fundend by #NEXTGENERATIONEU (NGEU) funded by the Ministry of University and Research (MUR), National Recovery and Resilience Plan (NRRP), project MNESYS (PE0000006) – A Multiscale integrated approach to the study of the nervous system in health and disease (DN. 1553 11.10.2022). S Morbelli was supported by a Grant from the Italian Ministry of University and Research (MIUR) (BANDO PRIN 2022 Prot. 2022WK7NHC)

**Compliance with Ethical Standards**

**Competing interest**. F Massa received speaker and consultant honoraria from Roche Diagnostic S.p.A and Eli Lilly S.p.A,; D Arnaldi received fees from Fidia, Bruno, Italfarmaco, Idorsia for lectures and board participation; S Morbelli received speaker Honoraria from G.E. Healthcare, Life Molecular imaging and Eli Lilly S.p.A.

**Informed consent and consent to publish.** This research involves human participants and was approved by the regional ethical committee (ref: RCR-2021–23671214). Informed consent was obtained from all participants. Anonymized data published per hospital rules for retrospective data collected during the clinical routine. All procedures followed the 1964 Helsinki declaration and its later amendments.

**Data availability statement.** Data is available on reasonable request from the corresponding author.

**Author Contributions:** All authors contributed to the study conception and design. All authors read and approved the final manuscript. **Conceptualization:** Denise Cerne, Federico Massa; **Methodology:** Federico Massa, Silvia Morbelli, Stefano Raffa; **Formal analysis and investigation:** Giulia Benvenuto, Denise Cerne, Anastasia Lechiara, Federico Massa, Emanuela Maria Mobilia, Silvia Morbelli, Stefano Raffa; **Writing - original draft preparation:** Denise Cerne, Federico Massa; **Writing - review and editing:** Luana Benedetti, Giulia Benvenuto, Denise Cerne, Federico Massa, Silvia Morbelli, Luca Roccatagliata, Dario Arnaldi; **Data Curation:** Giulia Benvenuto, Denise Cerne, Federico Massa; **Funding acquisition:** Luana Benedetti, Silvia Morbelli, Antonio Uccelli; **Supervision:** Luana Benedetti, Federico Massa; **Visualization:** Dario Arnaldi, Giacomo Bavestrello, Pietro Mattioli, Giacomo Rebella, Giampaola Pesce, Matteo Pardini, Antonio Uccelli, Flavio Villani; **Project administration:** Luana Benedetti

**References**

1. Abboud H, Probasco J, Irani SR, Ances B, Benavides DR, Bradshaw M, et al. Autoimmune encephalitis: proposed recommendations for symptomatic and long-term management. J Neurol Neurosurg Psychiatry. 2021;92:897–907.

2. Uy CE, Binks S, Irani SR. Autoimmune encephalitis: clinical spectrum and management. Pract Neurol. 2021;21:412–23.

3. Heine J, Duchow A, Rust R, Paul F, Prüß H, Finke C. Autoimmunenzephalitis – ein Update. Nervenarzt. 2023;94:525–37.

4. Titulaer MJ, McCracken L, Gabilondo I, Armangué T, Glaser C, Iizuka T, et al. Treatment and prognostic factors for long-term outcome in patients with anti-NMDA receptor encephalitis: an observational cohort study. Lancet Neurol. 2013;12:157–65.

5. Graus F, Titulaer MJ, Balu R, Benseler S, Bien CG, Cellucci T, et al. A clinical approach to diagnosis of autoimmune encephalitis. Lancet Neurol. 2016;15:391–404.

6. Lim J, Lee S, Moon J, Jun J, Kim T, Shin Y, et al. Development of the clinical assessment scale in autoimmune encephalitis. Ann Neurol. 2019;85:352–8.

7. Balu R, McCracken L, Lancaster E, Graus F, Dalmau J, Titulaer MJ. A score that predicts 1-year functional status in patients with anti-NMDA receptor encephalitis. Neurology. 2019;92.

8. Peng Y, Dai F, Liu L, Chen W, Yan H, Liu A, et al. Validation of the NEOS score in Chinese patients with anti-NMDAR encephalitis. Neurol Neuroimmunol Neuroinflamm. 2020;7.

9. Cai M-T, Lai Q-L, Zheng Y, Fang G-L, Qiao S, Shen C-H, et al. Validation of the Clinical Assessment Scale for Autoimmune Encephalitis: A Multicenter Study. Neurol Ther. 2021;10:985–1000.

10. Zhang Y, Tu E, Yao C, Liu J, Lei Q, Lu W. Validation of the Clinical Assessment Scale in Autoimmune Encephalitis in Chinese Patients. Front Immunol. 2021;12.

11. Liba Z, Kayserova J, Elisak M, Marusic P, Nohejlova H, Hanzalova J, et al. Anti-N-methyl-D-aspartate receptor encephalitis: the clinical course in light of the chemokine and cytokine levels in cerebrospinal fluid. J Neuroinflammation. 2016;13:55.

12. Ciano-Petersen NL, Cabezudo-García P, Muñiz-Castrillo S, Honnorat J, Serrano-Castro PJ, Oliver-Martos B. Current Status of Biomarkers in Anti-N-Methyl-D-Aspartate Receptor Encephalitis. Int J Mol Sci. 2021;22:13127.

13. Levraut M, Bourg V, Capet N, Delourme A, Honnorat J, Thomas P, et al. Cerebrospinal Fluid IL-17A Could Predict Acute Disease Severity in Non-NMDA-Receptor Autoimmune Encephalitis. Front Immunol. 2021;12.

14. Jeannin-Mayer S, André-Obadia N, Rosenberg S, Boutet C, Honnorat J, Antoine JC, et al. EEG analysis in anti-NMDA receptor encephalitis: Description of typical patterns. Clinical Neurophysiology. 2019;130:289–96.

15. Wesselingh R, Broadley J, Buzzard K, Tarlinton D, Seneviratne U, Kyndt C, et al. Electroclinical biomarkers of autoimmune encephalitis. Epilepsy & Behavior. 2022;128:108571.

16. Morbelli S, Djekidel M, Hesse S, Pagani M, Barthel H. Role of 18F-FDG-PET imaging in the diagnosis of autoimmune encephalitis. Lancet Neurol. 2016;15:1009–10.

17. Ances BM, Vitaliani R, Taylor RA, Liebeskind DS, Voloschin A, Houghton DJ, et al. Treatment-responsive limbic encephalitis identified by neuropil antibodies: MRI and PET correlates. Brain. 2005;128:1764–77.

18. Baumgartner A, Rauer S, Mader I, Meyer PT. Cerebral FDG-PET and MRI findings in autoimmune limbic encephalitis: correlation with autoantibody types. J Neurol. 2013;260:2744–53.

19. Bordonne M, Chawki MB, Doyen M, Kas A, Guedj E, Tyvaert L, et al. Brain 18F-FDG PET for the diagnosis of autoimmune encephalitis: a systematic review and a meta-analysis. Eur J Nucl Med Mol Imaging. 2021;48:3847–58.

20. Jha S, Nagaraj C, Mundlamuri R, Alladi S, Nashi S, Kenchaiah R, et al. FDG-PET in autoimmune encephalitis: Utility, pattern of abnormalities, and correlation with autoantibodies. Ann Indian Acad Neurol. 2022;25:1122.

21. Li G, Liu X, Yu T, Ren J, Wang Q. Positron emission tomography in autoimmune encephalitis: Clinical implications and future directions. Acta Neurol Scand. 2022;146:708–15.

22. Trevino-Peinado C, Arbizu J, Irimia P, Riverol M, Martínez-Vila E. Monitoring the Effect of Immunotherapy in Autoimmune Limbic Encephalitis Using 18F-FDG PET. Clin Nucl Med. 2015;40:e441–3.

23. Fisher RE, Patel NR, Lai EC, Schulz PE. Two Different 18F-FDG Brain PET Metabolic Patterns in Autoimmune Limbic Encephalitis. Clin Nucl Med. 2012;37:e213–8.

24. Yuan J, Guan H, Zhou X, Niu N, Li F, Cui L, et al. Changing Brain Metabolism Patterns in Patients With ANMDARE. Clin Nucl Med. 2016;41:366–70.

25. Liu L, Lyu Z, Li H, Bai L, Wan Y, Li P. Enhancing the clinical diagnosis of the acute and subacute phases of autoimmune encephalitis and predicting the risk factors: the potential advantages of 18F-FDG PET/CT. BMC Med Imaging. 2023;23:193.

26. Dai Y, Zhu Z, Tang Y, Xiao L, Liu X, Zhang M, et al. The clinical and predictive value of <scp> 18 F </scp> ‐ <scp>FDG PET</scp> / <scp>CT</scp> metabolic patterns in a clinical Chinese cohort with autoimmune encephalitis. CNS Neurosci Ther. 2024;30.

27. Guedj E, Varrone A, Boellaard R, Albert NL, Barthel H, van Berckel B, et al. Correction to: EANM procedure guidelines for brain PET imaging using [18F]FDG, version 3. Eur J Nucl Med Mol Imaging. 2022;49:2100–1.

28. Della Rosa PA, Cerami C, Gallivanone F, Prestia A, Caroli A, Castiglioni I, et al. A Standardized [18F]-FDG-PET Template for Spatial Normalization in Statistical Parametric Mapping of Dementia. Neuroinformatics. 2014;12:575–93.

29. Massa F, Filippi L, Benedetti L, Morbelli S, Nobili F. FDG PET Unveils the Course of Paraneoplastic Cerebellar Degeneration. Clin Nucl Med. 2021;46:e327–8.

30. Cistaro A, Valentini MC, Chiò A, Nobili F, Calvo A, Moglia C, et al. Brain hypermetabolism in amyotrophic lateral sclerosis: a FDG PET study in ALS of spinal and bulbar onset. Eur J Nucl Med Mol Imaging. 2012;39:251–9.

31. Perani D, Della Rosa PA, Cerami C, Gallivanone F, Fallanca F, Vanoli EG, et al. Validation of an optimized SPM procedure for FDG-PET in dementia diagnosis in a clinical setting. Neuroimage Clin [Internet]. 2014 [cited 2020 Jan 7];6:445–54. Available from: http://www.ncbi.nlm.nih.gov/pubmed/25389519

32. Buchert R, Wilke F, Chakrabarti B, Martin B, Brenner W, Mester J, et al. Adjusted Scaling of FDG Positron Emission Tomography Images for Statistical Evaluation in Patients With Suspected Alzheimer’s Disease. Journal of Neuroimaging [Internet]. 2005;15:348–55. Available from: https://doi.org/10.1111/j.1552-6569.2005.tb00335.x

33. Lieberman MD, Cunningham WA. Type I and Type II error concerns in fMRI research: re-balancing the scale. Soc Cogn Affect Neurosci. 2009;4:423–8.

34. Massa F, Grisanti S, Brugnolo A, Doglione E, Orso B, Morbelli S, et al. The role of anterior prefrontal cortex in prospective memory: an exploratory FDG-PET study in early Alzheimer’s disease. Neurobiol Aging. 2020;96:117–27.

35. Kreshpa W, Raffa S, Girtler N, Brugnolo A, Mattioli P, Orso B, et al. Limbic Network Derangement Mediates Unawareness of Apathy in Mild Cognitive Impairment due to Alzheimer’s Disease: Clues from [18F]FDG PET Voxel-Wise Analysis. Journal of Alzheimer’s Disease. 2024;101:475–85.

36. Nobili F, Schmidt R, Carriò I, Frisoni GB. Brain FDG-PET: clinical use in dementing neurodegenerative conditions. Eur J Nucl Med Mol Imaging. 2018;45:1467–9.

37. Johnston R, Jones K, Manley D. Confounding and collinearity in regression analysis: a cautionary tale and an alternative procedure, illustrated by studies of British voting behaviour. Qual Quant. 2018;52:1957–76.

38. Brunet de Courssou J-B, Castilla-Lievre MA, Maillot J, Brechemier M-L, Ohlmann C, Sallansonnet-Froment M, et al. Autoimmune cerebellar hypermetabolism: Report of three cases and literature overview. Rev Neurol (Paris) [Internet]. 2022;178:337–46. Available from: https://www.sciencedirect.com/science/article/pii/S0035378721007013

39. Massa F, Filippi L, Benedetti L, Morbelli S, Nobili F. FDG PET Unveils the Course of Paraneoplastic Cerebellar Degeneration: A Semiquantitative Analysis. Clin Nucl Med [Internet]. 2021 [cited 2021 Dec 18];46:E327–8. Available from: https://journals.lww.com/nuclearmed/Fulltext/2021/06000/FDG_PET_Unveils_the_Course_of_Paraneoplastic.31.aspx

40. Lancaster E, Dalmau J. Neuronal autoantigens—pathogenesis, associated disorders and antibody testing. Nat Rev Neurol. 2012;8:380–90.

41. Hunter D, Petit-Pedrol M, Fernandes D, Bénac N, Rodrigues C, Kreye J, et al. Converging synaptic and network dysfunctions in distinct autoimmune encephalitis. EMBO Rep. 2024;25:1623–49.

42. Gibson LL, McKeever A, Coutinho E, Finke C, Pollak TA. Cognitive impact of neuronal antibodies: encephalitis and beyond. Transl Psychiatry. 2020;10:304.

43. Zhang W, Wang J, Fan L, Zhang Y, Fox PT, Eickhoff SB, et al. Functional organization of the fusiform gyrus revealed with connectivity profiles. Hum Brain Mapp. 2016;37:3003–16.

44. Chen C, Liu Z, Zuo J, Xi C, Long Y, Li MD, et al. Decreased Cortical Folding of the Fusiform Gyrus and Its Hypoconnectivity with Sensorimotor Areas in Major Depressive Disorder. J Affect Disord. 2021;295:657–64.

45. Palejwala AH, O’Connor KP, Milton CK, Anderson C, Pelargos P, Briggs RG, et al. Anatomy and white matter connections of the fusiform gyrus. Sci Rep. 2020;10:13489.

46. Chang EF, Raygor KP, Berger MS. Contemporary model of language organization: an overview for neurosurgeons. J Neurosurg. 2015;122:250–61.

47. Xu J, Wang J, Fan L, Li H, Zhang W, Hu Q, et al. Tractography-based Parcellation of the Human Middle Temporal Gyrus. Sci Rep. 2015;5:18883.

48. Ohnishi H, Matsuoka K, Takahashi M, Yoshikawa H, Minami A, Ueda K, et al. Associations of demyelination in the right middle temporal gyrus and right praecuneus with visuospatial cognitive dysfunction in Alzheimer’s disease. Psychogeriatrics. 2025;25.

49. Uddin LQ, Yeo BTT, Spreng RN. Towards a Universal Taxonomy of Macro-scale Functional Human Brain Networks. Brain Topogr. 2019;32:926–42.

50. Correction: Utevsky et al., “Precuneus Is a Functional Core of the Default-Mode Network.” The Journal of Neuroscience. 2016;36:12066–8.

51. Mohan A, Roberto AJ, Mohan A, Lorenzo A, Jones K, Carney MJ, et al. The Significance of the Default Mode Network (DMN) in Neurological and Neuropsychiatric Disorders: A Review. Yale J Biol Med. 2016;89:49–57.

52. Zanão TA, Lopes TM, de Campos BM, Yasuda CL, Cendes F. Patterns of default mode network in temporal lobe epilepsy with and without hippocampal sclerosis. Epilepsy & Behavior. 2021;121:106523.

53. González Otárula KA, Schuele S. Networks in Temporal Lobe Epilepsy. Neurosurg Clin N Am. 2020;31:309–17.

54. Sachdev PS, Wang H. The default mode network, depression and Alzheimer’s disease. Int Psychogeriatr. 2022;34:675–8.

55. Schimmelpfennig J, Topczewski J, Zajkowski W, Jankowiak-Siuda K. The role of the salience network in cognitive and affective deficits. Front Hum Neurosci. 2023;17.

56. Karunakaran S, Rollo MJ, Kim K, Johnson JA, Kalamangalam GP, Aazhang B, et al. The interictal mesial temporal lobe epilepsy network. Epilepsia. 2018;59:244–58.

57. Powell HWR, Parker GJM, Alexander DC, Symms MR, Boulby PA, Wheeler-Kingshott CAM, et al. Hemispheric asymmetries in language-related pathways: A combined functional MRI and tractography study. Neuroimage. 2006;32:388–99.

58. Bressler SL, Menon V. Large-scale brain networks in cognition: emerging methods and principles. Trends Cogn Sci. 2010;14:277–90.

59. Zhuo C, Zhu J, Wang C, Qu H, Ma X, Qin W. Different spatial patterns of brain atrophy and global functional connectivity impairments in major depressive disorder. Brain Imaging Behav. 2017;11:1678–89.

60. Rangarajan V, Hermes D, Foster BL, Weiner KS, Jacques C, Grill-Spector K, et al. Electrical Stimulation of the Left and Right Human Fusiform Gyrus Causes Different Effects in Conscious Face Perception. Journal of Neuroscience. 2014;34:12828–36.

61. Rissanen E, Carter K, Cicero S, Ficke J, Kijewski M, Park M-A, et al. Cortical and Subcortical Dysmetabolism Are Dynamic Markers of Clinical Disability and Course in Anti-LGI1 Encephalitis. Neurology - Neuroimmunology Neuroinflammation. 2022;9:e1136.

62. Kvam KA, Stahl J-P, Chow FC, Soldatos A, Tattevin P, Sejvar J, et al. Outcome and Sequelae of Autoimmune Encephalitis. Journal of Clinical Neurology. 2024;20:3.

63. Heine J, Prüss H, Kopp UA, Wegner F, Then Bergh F, Münte T, et al. Beyond the limbic system: disruption and functional compensation of large-scale brain networks in patients with anti-LGI1 encephalitis. J Neurol Neurosurg Psychiatry. 2018;89:1191–9.

64. Leypoldt F, Buchert R, Kleiter I, Marienhagen J, Gelderblom M, Magnus T, et al. Fluorodeoxyglucose positron emission tomography in anti-N-methyl-D-aspartate receptor encephalitis: distinct pattern of disease. J Neurol Neurosurg Psychiatry. 2012;83:681–6.

65. Guerin J, Watson RE, Carr CM, Liebo GB, Kotsenas AL. Autoimmune epilepsy: findings on MRI and FDG-PET. Br J Radiol. 2019;92.

66. Kunchok A, McKeon A, Zekeridou A, Flanagan EP, Dubey D, Lennon VA, et al. Autoimmune/Paraneoplastic Encephalitis Antibody Biomarkers: Frequency, Age, and Sex Associations. Mayo Clin Proc. 2022;97:547–59.

67. Massa F, Franciotta D, Grisanti S, Roccatagliata L, Morbelli S, Beltramini S, et al. Intravenous immunoglobulin bridging to rituximab in NMDAR encephalitis patients non-responders to first-line treatments. Neurological Sciences. 2022;43:6441–7.

68. Bastiaansen AEM, van Steenhoven RW, de Bruijn MAAM, Crijnen YS, van Sonderen A, van Coevorden-Hameete MH, et al. Autoimmune Encephalitis Resembling Dementia Syndromes. Neurology - Neuroimmunology Neuroinflammation. 2021;8:e1039.

69. McKeon GL, Robinson GA, Ryan AE, Blum S, Gillis D, Finke C, et al. Cognitive outcomes following anti-N-methyl-D-aspartate receptor encephalitis: A systematic review. J Clin Exp Neuropsychol. 2018;40:234–52.

70. Dodich A, Cerami C, Iannaccone S, Marcone A, Alongi P, Crespi C, et al. Neuropsychological and FDG-PET profiles in VGKC autoimmune limbic encephalitis. Brain Cogn. 2016;108:81–7.

71. Galioto R, Grezmak T, Swetlik C, Abbatemarco JR, Titulaer MJ, Finke C, et al. Neuropsychological Testing in Autoimmune Encephalitis. Neurol Neuroimmunol Neuroinflamm. 2024;11.

72. Blumenfeld H, McNally KA, Vanderhill SD, Paige AL, Chung R, Davis K, et al. Positive and Negative Network Correlations in Temporal Lobe Epilepsy. Cerebral Cortex. 2004;14:892–902.

73. Siclari F, Prior JO, Rossetti AO. Ictal cerebral positron emission tomography (PET) in focal status epilepticus. Epilepsy Res. 2013;105:356–61.

74. REBELLA G, CERNE D, BENEDETTI L, MORBELLI S, RESAZ M, UCCELLI A, et al. FDG-PET and ASL MRI identify largely overlapping hypermetabolic and hyperperfusion changes in limbic autoimmune encephalitis. The Quarterly Journal of Nuclear Medicine and Molecular Imaging. 2024;68.

75. Cotta Ramusino M, Massa F, Festari C, Gandolfo F, Nicolosi V, Orini S, et al. Diagnostic performance of molecular imaging methods in predicting the progression from mild cognitive impairment to dementia: an updated systematic review. Eur J Nucl Med Mol Imaging. 2024;51:1876–90.

76. Nobili F, Arbizu J, Bouwman F, Drzezga A, Agosta F, Nestor P, et al. European Association of Nuclear Medicine and European Academy of Neurology recommendations for the use of brain 18 F‐fluorodeoxyglucose positron emission tomography in neurodegenerative cognitive impairment and dementia: Delphi consensus. Eur J Neurol. 2018;25:1201–17.

**Table 1. Demographic, clinical and treatment data**

| Demographic information (n=22) | |
| --- | --- |
| Female, N (%)  Age, mean ± SD | 12 (54.5)  56.3±20.3 |
| Diagnosis according to Graus criteria (n=22) | |
| Definite limbic AE, N (%)  Definite NMDAR AE, N (%)  Probable seronegative AE, N (%) | 11 (50)  4 (18.2)  7 (31.8) |
| Auto-antibodies (n=22) | |
| LGI1, N (%)  NMDAR, N (%)  CASPR2, N (%)  LGI1 and CASPR2, N (%)  Onconeural, N (%)  Ma2, N (%)  Yo, N (%)  Tr/DNER, N (%)  Seronegative, N (%) | 6 (27.3)  4 (18.2)  1 (4.5)  1 (4.5)  3 (13.6)  1 (4.5)  1 (4.5)  1 (4.5)  7 (31.8) |
| CSF findings (n=18) | |
| Normal, N (%)  Pleocytosis, N (%)  Elevated protein levels, N (%)  Oligoclonal bands, N (%) | 6 (33.3)  7 (21.8)  5 (22.7)  10 (55.5) |
| Brain MRI (n=22) | |
| Presence of abnormalities, N (%) | 11 (50) |
| Symptoms at onset (n=22) | |
| Seizure, N (%)  Psychiatric symptoms, N (%)  Cognitive impairment, N (%)  Other, N (%) | 13 (59.1)  11 (50)  11 (50)  13 (59.1) |
| Presence of neoplasia (n=22) | |
| Yes, N (%) | 9 (40.9) |
| Therapy (n=22) | |
| *1*^st^ *line, N (*%)  Steroid, N (%)  IVIG, N (%)  Steroid+PEX, N (%)  Steroid+IVIG, N (%)  Steroid+PEX+IVIG, N (%)  *2*^nd^ *line, N* (%)  Rituximab, N (%)  Azathioprine, N (%) | 22 (100)  3 (13.6)  5 (22.7)  1 (4.5)  7 (31.8)  6 (27.3)  10 (45.5)  9 (41)  1 (4.5) |
| Relapse (n=22) | |
| Yes, N (%) | 9 (40.9) |
| CASE score (n=22) | |
| BS timepoint, mean±SD  FU timepoint, mean±SD | 4.4±2.3  2.5±0.59 |
| mRS score (n=22) | |
| BS timepoint, mean±SD  FU timepoint, mean±SD | 3.1±0.66  2.0±0.76 |

**Table 2.** **Regions with significant metabolic differences between BS and FU (F-contrast)**

| **Brain areas involved in VOI-A** | | | | | | | |
| --- | --- | --- | --- | --- | --- | --- | --- |
| **Cluster p (FEW-corr)** | **Cluster extension (number of voxel)** | **Cluster p (unc)** | **Cluster peaks coordinates**  **x y z** | | | **Cortical region** | **BA** |
| 0.037 | 756 | 0.003 | 4.71 | 3.15 | -7.96 | R anterior cingulate | 25 |
|  |  |  | 4.59 | 2.28 | 0.97 | R caudate head | - |
|  |  |  | 2.81 | 4.67 | -4.24 | R anterior cingulate | 25 |
|  |  |  | -6.54 | 2.16 | 2.57 | L caudate head | - |
|  |  |  | 6.3 | -6.05 | 9.22 | R thalamus | - |
|  |  |  | -2.93 | -7.7 | 7.1 | L thalamus | - |
|  |  |  | 25.02 | 13.72 | -9.21 | R amygdala | - |
|  |  |  | -2,99 | -9,91 | 10,49 | L thalamus | **-** |
|  |  |  | 12.13 | -5.86 | -12.29 | R parahippocampal G | 34 |
| **Brain areas involved in VOI-B** | | | | | | | |
| 0.004 | 1160 | 0.003 | 11.37 | -59.0 | 31.31 | R precuneus | 7 |
|  |  |  | 45.0 | -53.18 | 9.01 | R superior temporal G | 39 |
|  |  |  | 39.12 | -62.87 | 31.41 | R middle temporal G | 39 |
|  |  |  | 44.74 | -56.96 | 28.46 | R superior temporal G | 39 |
|  |  |  | 13.25 | -64.25 | 27.24 | R precuneus | 31 |
|  |  |  | 9.41 | -65.28 | 37.89 | R precuneus | 7 |
|  |  |  | 44.64 | -50.38 | 38.1 | R inferior parietal Lo | 40 |
|  |  |  | 52.12 | -46.17 | 33.22 | R supramarginal G | 40 |
|  |  |  | 27.93 | -70.79 | 35.88 | R precuneus | 19 |
|  |  |  | 31.75 | -58.93 | 29.86 | R middle temporal G | 39 |
|  |  |  | 33.62 | -71.64 | 25.08 | R superior occipital G | 19 |
|  |  |  | 41.22 | -66.56 | 11.28 | R middle occipital G | 19 |

Peak coordinates and cortical regions in each cluster are ordered downward from the highest Z-score peak. Abbreviations: BS, baseline; FU, follow up; BA, Brodmann area; R, right; L, left; G, gyrus; Lo, lobule

**Table 3. Relative hypermetabolic (VOI-A) and hypometabolic (VOI-B) regions in BS compared to FU**

| **Relative hypermetabolic areas at BS (VOI-A)** | | | | | | | |
| --- | --- | --- | --- | --- | --- | --- | --- |
| **Cluster p (FEW-corr)** | **Cluster extension (number of voxel)** | **Cluster p (unc)** | **Cluster peaks coordinates**  **x y z** | | | **Cortical region** | **BA** |
| 0.016 | 854 | 0.003 | 4.71 | 3.15 | -7.96 | R anterior cingulate | 25 |
|  |  |  | 4.59 | 2.28 | 0.97 | R caudate head | - |
|  |  |  | 2.81 | 4.67 | -4.24 | R anterior cingulate | 25 |
|  |  |  | -6.54 | 2.16 | 2.57 | L caudate head | - |
|  |  |  | 6.3 | -6.05 | 9.22 | R thalamus | - |
|  |  |  | -2.93 | -7.7 | 7.1 | L thalamus | - |
|  |  |  | 25.02 | 13.72 | -9.21 | R amygdala | - |
|  |  |  | -2,99 | -9,91 | 10,49 | L thalamus | **-** |
|  |  |  | 12.13 | -5.86 | -12.29 | R parahippocampal G | 34 |
| **Relative hypometabolic areas at BS (VOI-B1)** | | | | | | | |
| 0.019 | 817 | 0.003 | 51.02 | -26.03 | -18.95 | R inferior temporal G | 20 |
|  |  |  | 54.58 | -25.23 | -8.0 | R middle temporal G | 21 |
|  |  |  | 43.6 | -31.58 | -19.6 | R fusiform G | 20 |
|  |  |  | 43.45 | -54.64 | -14.58 | R fusiform G | 37 |
|  |  |  | 48.99 | -41.97 | -9.68 | R fusiform G | 37 |
|  |  |  | 45.36 | -39.57 | -14.92 | R fusiform G | 37 |
|  |  |  | 50.95 | -44.84 | -18.93 | R fusiform G | 37 |
|  |  |  | 54.69 | -37.23 | -19.95 | R fusiform G | 20 |
| **Relative hypometabolic areas at BS (VOI-B2)** | | | | | | | |
| 0 | 1701 | 0.003 | 11.37 | -59.0 | 31.31 | R precuneus | 7 |
|  |  |  | 45.0 | -53.18 | 9.01 | R superior temporal G | 39 |
|  |  |  | 39.12 | -62.87 | 31.41 | R middle temporal G | 39 |
|  |  |  | 44.74 | -56.96 | 28.46 | R superior temporal G | 39 |
|  |  |  | 13.25 | -64.25 | 27.24 | R precuneus | 31 |
|  |  |  | 9.41 | -65.28 | 37.89 | R precuneus | 7 |
|  |  |  | 44.64 | -50.38 | 38.1 | R inferior parietal Lo | 40 |
|  |  |  | 52.12 | -46.17 | 33.22 | R supramarginal G | 40 |
|  |  |  | 27.93 | -70.79 | 35.88 | R precuneus | 19 |
|  |  |  | 31.75 | -58.93 | 29.86 | R middle temporal G | 39 |
|  |  |  | 33.62 | -71.64 | 25.08 | R superior occipital G | 19 |
|  |  |  | 41.22 | -66.56 | 11.28 | R middle occipital G | 19 |

Significant brain areas resulting from comparison of brain metabolism between BS and FU clusters (T-test), considering a priori FU as normalizing the picture. Relative hypermetabolism in VOI-A; relative hypometabolism in VOI-B (both B1 and B2). Peak coordinates and cortical regions in each cluster are ordered downward from the highest Z-score peak. Abbreviations: BS, baseline; FU, follow up; BA, Brodmann area; R, right; L, left; G, gyrus; Lo, lobule

**Figure legends**

**
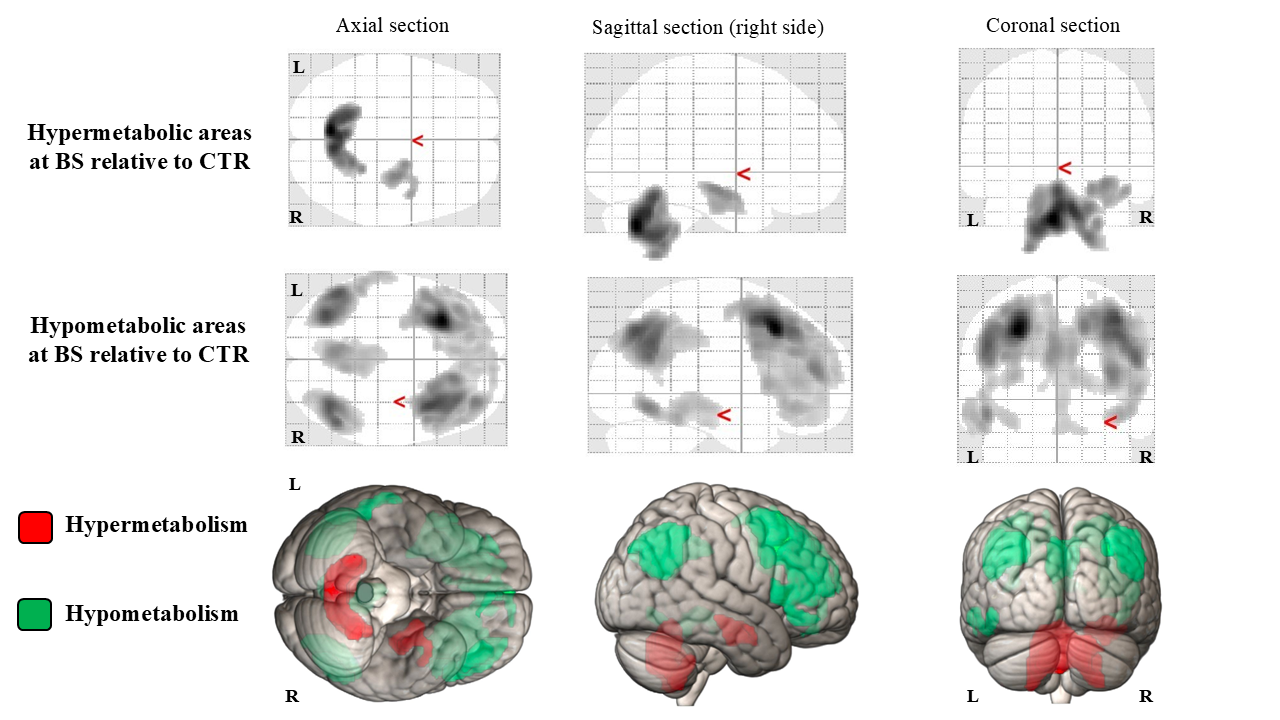
**

**Figure 1.** In the first two lines, T-maps as seen from the axial, sagittal and coronal sections of the 2 VOI (i.e., hypermetabolic and hypometabolic areas) from the direct comparison between BS and CTR groups (height threshold of p<0.002, uncorrected for multiple comparisons at peak level; p<0.033 FEW for multiple comparisons at cluster level). In the lower part, 3D rendering of the same areas (i.e., red for hypertabolism and green for hypometabolism) in the MNI reference atlas (using MRIcroGL software, https://www.nitrc.org/projects/mricrogl). Abbreviations: VOI – volume of interest; BS – baseline; CTR – control group; R – right; L - left


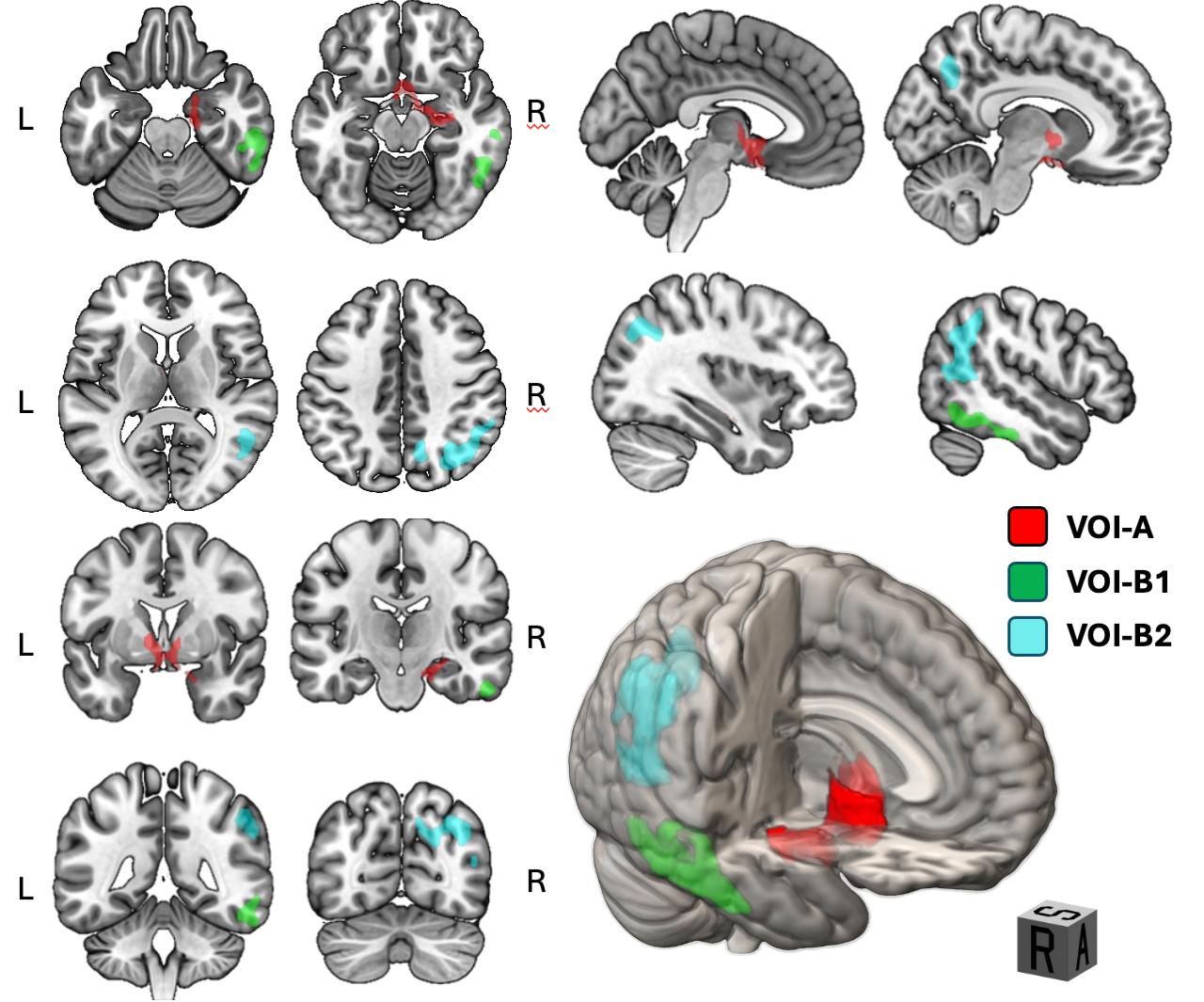


**Figure 2.** The two-dimensional representation (axial, coronal and sagittal cuts) and three-dimensional rendering show VOI-A in red, VOI-B1 in green and VOI-B2 in light blue superimposed on the MNI reference atlas (using MRIcroGL software, https://www.nitrc.org/projects/mricrogl). In the 3D rendering, mid-line sagittal and coronal cuts were used to enhance the visualization of the VOIs. Abbreviations: VOI - volume of interest; L – left; R – right; P – posterior; S – superior; A – anterior


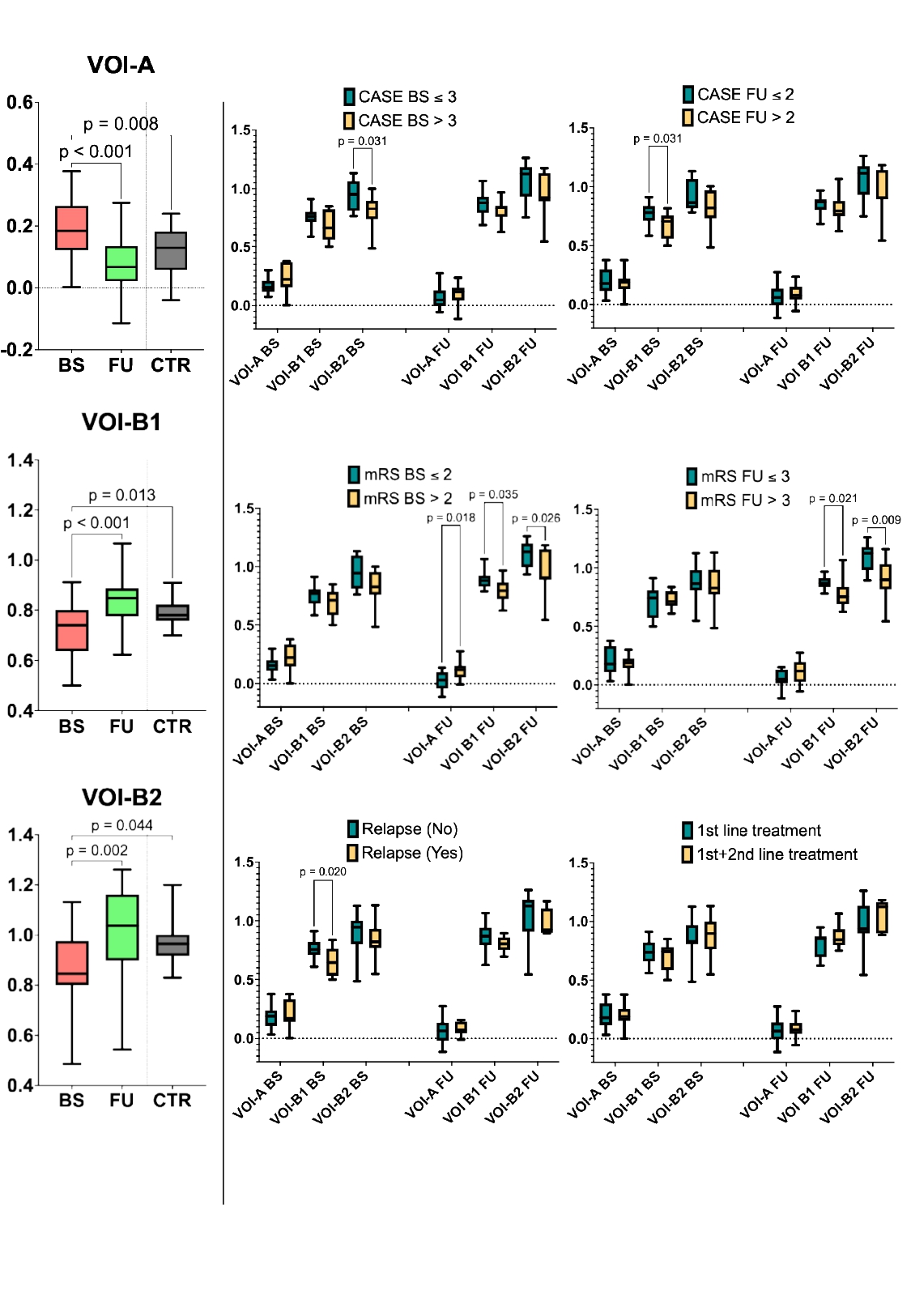


**Figure 3.** On the left, VOI-based analyses and post-hoc analysys in the group comparisons (BS, FU, CTR). The absence of a significant difference between VOI values of patients at FU and those of the CTR group supported a trend toward metabolic normalization after treatment. On the right, distinct VOIs significantly differed in AE patients grouped according to dichotomized clinical outcomes. Abbreviations: VOI - volume of interest; BS – baseline; FU – follow-up; CTR – control group
