# Supplementary material for: Longitudinal changes in [18F]FDG PET brain metabolism as a prognostic marker in autoimmune encephalitis
Source: Eur J Nucl Med Mol Imaging. 2025 Aug 25;53(2):1183–97. doi: 10.1007/s00259-025-07526-2 (PMC12830435; doi:10.1007/s00259-025-07526-2)
Supplement: Supplementary file 2 — (DOCX 17.8 KB) [file 259_2025_7526_MOESM2_ESM.docx]

Ref. n. EJNM-D-25-01104

To Professor Ignasi Carrio, MD, FEBNM, FRSC, FRCP

Autonomous University of Barcelona, Spain

Genoa, July 28th, 2025

Dear Editor-in-Chief,

on behalf of all the co-authors, we thank and acknowledge the editors and reviewers for the positive comments and the criticisms aimed to improve the manuscript. We prepared a revised version of the manuscript following their suggestions. The modified parts in response to reviewers are highlighted in yellow while adjustments to the language style are crossed out. We also uploaded an unmarked version of the manuscript for your convenience.

Specifically, we have:

- enriched the methods and materials section by better describing control group and voxel-base analysis
- revised the regression models by adding the ‘sex’ in the full model with the main clinical variables at baseline (age, sex, CASE BS, mRS BS) and VOI BS values
- clarified the analytical decision to select volumes of interest (VOIs) containing at least 100 voxels and voxel-level thresholds to facilitate methodological understanding
- we conducted ROC analyses to assess predictive accuracy and identify optimal VOI‑B1 BS thresholds —based on the highest likelihood ratios—for relapse and CASE at follow‑up, guided by our linear models in which VOI‑B1 proved the strongest predictor of these outcomes. This with the aim of exploring practical implications for clinical translation. The resulting ROI curves and cut-off values are now displayed in the

in Supplementary Figure 1s of the revised manuscript

- discussed the limitations of the study - especially addressing the sample size and the lack of a formal neuropsychological assessment - in more detail

We have also included a response to the reviewers in addition to making the changes described above in the manuscript.

We hope the revised version is now acceptable for publication in European Journal of Nuclear Medicine and Molecular Imaging.

Sincerely,

Federico Massa, MD, PhD

Department of Neuroscience, Rehabilitation, Ophthalmology, Genetics, Maternal and Child Health (DINOGMI), University of Genoa,

Largo Daneo 3, 16132 Genoa (Italy)

e-mail: fedemassa88@gmail.com
